# Tumor grade-associated genomic mutations in Chinese patients with non-small cell lung cancer

**DOI:** 10.3389/fonc.2023.1119575

**Published:** 2023-03-20

**Authors:** Yang Wang, Shilei Qin, Yuepei Liang, Ling Yan, Min Zheng, Yanwu Zeng, Leilei Lu

**Affiliations:** ^1^ Department of Thoracic Surgery, Affiliated Hospital of Guilin Medical University, Guilin, China; ^2^ Operations Department, Shanghai OrigiMed Co., Ltd., Shanghai, China

**Keywords:** lung cancer, tumor grade, genomic mutations, Epstein-Barr virus, pathway analysis

## Abstract

**Background:**

Lung cancer is the most prevalent cancer worldwide and accounts for approximately 20% of cancer-related death in China every year. High-grade lung cancer poses a significant threat to patients, and developing a novel treatment for these patients requires an understanding of its underlying mechanism.

**Methods:**

Chinese patients with lung cancer were enrolled. The tumor samples were collected by surgery or puncture and applied for next-generation sequencing. A panel of pan-cancer genes was targeted, and the sequencing depth was set to over 1,000 to improve the sensitivity of detecting mutations. Short-length mutations (substitution, insertion, and deletion), copy number variation, and gene fusion were called. Gene mutations were compared between low-grade, middle-grade, and high-grade tumors using Fisher’s exact test. The enriched pathways in each grade of tumors were also inferred.

**Results:**

The study included 173 Chinese patients with non-small cell lung cancer, of whom 98 (56.6%) patients were female and 75 (43.4%) were male, with a mean age of 56.8 years. All patients were microsatellite stable; 66.4% were at the early stages (Stages 0, I, and II) with a tumor mutational burden of approximately 2.5 (confidence interval = [0, 48.3]). Compared to low-grade tumors, high-grade tumors had a significantly higher percentage of mutations in *TP53* (75.9% *vs* 34.4%, *p* = 1.86e-3) and *PIK3CA* (24.1% *vs*. 0%, *p* = 3.58e-3). Pathway analysis found that high-grade tumors were enriched with mutations in bacterial invasion of epithelial cells (31% *vs*. 0%, *p* = 5.8e-4), Epstein–Barr virus infection (79.3% *vs*. 37.5%, *p* = 1.72e-3), and the Wnt signaling pathway (75.9% *vs*. 34.4%, *p* = 1.91e-3). High-grade tumors had a significantly higher tumor mutational burden than low-grade tumors (*p*-value = 0.0017). However, actionable mutations with high-level evidence were lower in high-grade tumors.

**Conclusion:**

Patients with high-grade tumors from lung cancer may be more affected by bacteria and Epstein–Barr virus than low-grade tumors. High-grade tumors were specially mutated in *TP53* and *PIK3CA* and may benefit more from immunotherapy. Further research on the underlying mechanism of high-grade lung cancer is necessary to develop new therapeutic options. Lung cancer, tumor grade, genomic mutations, Epstein–Barr virus, pathway analysis

## Introduction

Lung cancer is a pervasive form of cancer worldwide, accounting for approximately 20% of cancer-related death in China alone (1). Non-small cell lung cancer (NSCLC) represents approximately ~80% of lung cancer cases (2) and its average overall 5-year survival rate is 32.8%, varying widely from 20.3% to 64.7% among patients (3). Given the diverse feature of NSCLC, further subtyping is crucial for customized treatments.

Tumor grade is a common biomarker used to subtype cancers, and the World Health Organization classification provides a grading system for NSCLC (4). For example, solid or micropapillary predominant adenocarcinoma is classified as high grade, acinar and papillary adenocarcinoma as intermediate grade, and lepidic adenocarcinoma as low grade; large cell neuroendocrine carcinoma is always categorized as high grade. High-grade NSCLC has a median overall survival of 21.6 months (95% CI, 6.8–77.2), which is significantly shorter than other tumors (5). Despite its severity, there are no special treatments for patients with high-grade NSCLC. Targeted therapy and immunotherapy have shown promise for a specific patient group, but knowledge of gene mutations in high-grade NSCLC is limited. Thus, treating patients with high-grade tumors using these techniques remains a challenge, and identifying mutations and understanding their potential mechanisms are crucial.

To address this issue, we conducted a study that enrolled 173 patients with NSCLC, of whom 168 patients had qualified samples for high-depth panel sequencing. We displayed and compared gene mutation profiling between different grades of NSCLC, and functional enrichment analysis revealed the pathway preference of gene mutations for each grade. Additionally, we extracted mutational signatures for each grade of NSCLC and compared them to a known mutational signature database.

## Patients and methods

### Patients and tumor sample collection

This study was approved by the Ethics Committee of the Affiliated Hospital of Guilin Medical University. The patient inclusion criteria were as follows: (1) patients were diagnosed with NSCLC; (2) patients have qualified paired tumor and blood samples for sequencing; and (3) patients have provided written informed consent. The patient exclusion criteria were as follows: (1) paired samples were successfully sequenced; and (2) the sequencing results had identified mutations. Finally, we enrolled 173 Chinese patients (GMU cohort). The staging system most often used for NSCLC is the 9th edition of the American Joint Committee on Cancer (AJCC) TNM system. Tumor samples were collected by either surgery or puncture and immediately fixed with 4% formalin buffer at 4°C for 24 h. The fixed samples were then embedded in paraffin wax for storage.

We also downloaded a public cohort (MSK cohort) (6) from cBioportal (http://www.cbioportal.org) for comparison against our cohort. The MSK cohort contained 247 patients with NSCLC.

### Pathologic examination

All samples were classified into low, middle, and high grades with histological classification and grade according to the fourth edition of the World Health Organization classification (4) by two expert pathologists.

### Library construction and sequencing

FFPE (formalin-fixed paraffin-embedded) tissues were sliced into 4-µm sections. One section of the FFPE sample was dewaxed on the glass slide in a 60°C oven for 1 h and washed with xylene for 15 min. The samples were then processed with gradient concentrations (100%, 95%, 85%, and 75%) of alcohol, cooked with pressure for 4 min, and immersed in cold water for 10 min. These samples were then stained with hematoxylin and eosin. The stained slice underwent a pathologist review to ensure the quality requirements including stained area exceeding 1 cm^2^, 20% nucleated cellularity, and 20% tumor content. After the sample passed the quality check, 10 unstained FFPE sections were used for sequencing library construction. The library construction and sequencing were performed in the CLIA/CAP-compliant Molecular Diagnostics Service laboratory of Shanghai Origimed Co., Ltd. The detailed procedure had been described in our previous works (7–9). Briefly, typically 50–250 ng of double-stranded DNA was fragmented to ~250 bp by sonication. The KAPA Hyper Prep Kit (KAPA Biosystems) was used for end repair. The following procedures included dA addition, ligation, PCR amplification, and Qubit (Thermofisher Corporation) quality assessment. Samples yielding >40 ng of extracted DNA or 500 ng of sequencing library were further used. A custom-targeted panel of 450 cancer-related genes (Yuansuo^®^, Origimed incorporation, Shanghai, China) was specifically amplified. Unique molecular identifiers were added to each sonication-shuffled segments before library amplification. Libraries were diluted to 1.05 nM and subsequently sequenced on Illumina NextSeq 500. Paired primers were extended from both ends of targeting sequences for 75 bp. To improve the sensitivity of detecting mutations, the average sequencing depth is over 1000×. For each patient, white blood cells were extracted from paired blood samples. The same library construction and sequencing procedure were applied for white blood cells. 

### Mutation calling and bioinformatics analysis

The output reads from Illumina NextSeq 500 sequencer were mapped to the human genome (GPRC37) with bwa-mem (version 0.7.9a) and recalibrated with GATK BaseRecalibrator (version 3.8). The mapped reads were deduplicated according to the unique molecular identifiers. Mutect2 (10) and varscan (11) were used to call short nucleotide mutations including single-nucleotide variants and small indels as well. Somatic mutations were annotated and filtered out for possible germline mutations from the known single-nucleotide polymorphism database (ESP6500, 1000 Genomes, gnomAD, and ExAC). Germline mutations were also obtained from sequencing white blood cells for each patient. CNVkit (12) and an in-house pipeline (13) were used to call copy number variation and gene fusion. Tumor mutational burden (TMB) was calculated as the number of mutations dividing the effective coverage length in millions. MutSigCV was used to identify significantly mutated genes, which compares gene mutation frequency by considering mutation biases including gene length and cancer types (14).

Mutational signature analysis was performed for different grades of tumors using the R package “MutationalPatterns” (v3.4.1). First, 96 single-nucleotide mutation contexts were decomposed into components with negative-matrix factorization. The major components were compared to the known COSMIC mutational signature database (https://cancer.sanger.ac.uk/cosmic). The analysis was performed on the R computation platform (v4.05).

A heatmap was generated with the R package “HeatmapComplex” (v2.15.1), bar and scatter plots were generated with the R package “ggpubr” (v0.5.0), and a Sankey plot was generated with the R package “ggalluvial” (v0.12.3).

### Statistical analysis

Categorical and scale variables were compared with Fisher’s exact test and Mann–Whitney *U* test, respectively. The mutation status is defined as “mutated” when a gene or pathway has any mutation in them; otherwise, it is defined as “wild”. The mutation status for each gene and gene pathway was treated as a categorical variable. Benjamin and Hochberg’s method was used to correct the errors from multiple tests. *p-*values below 0.05 were defined as statistically significant. All the analysis was performed on the R computation platform (v4.05).

## Results

### Clinical characteristics

This study enrolled 173 Chinese patients (GMU cohort) with lung cancer, of whom 98 (56.6%) patients were female and 75 (43.4%) were male (**Table 1**). The mean age of these patients was 56.8 years. None of the patients were microsatellite instable. Most of them (66.4%) were at the early stage (Stages 0, I, and II). Patients at advanced stages (Stages III and IV) accounted for 28.3%. The median TMB was 2.5 Mut/Mbp (confidence interval = [0, 48.3]). Of the 173 patients, 29 (16.8%) had high-grade tumors, 34 (19.7%) had middle-grade tumors, and 32 (18.5%) had low-grade tumors.

**Table 1:** Clinical characteristics.

	Overall (*N* = 173)
Age	
Mean (SD)	56.8 (11.7)
Median [Min, Max]	56.0 [24.0, 85.0]
Sex	
Female	98 (56.6%)
Male	75 (43.4%)
Stage	
0	12 (6.9%)
I	86 (49.7%)
II	17 (9.8%)
III	21 (12.1%)
IV	28 (16.2%)
Missing	9 (5.2%)
MSI	
MSS	173 (100%)
TMB	
Mean (SD)	4.95 (7.06)
Median [Min, Max]	2.50 [0, 48.3]
Grade	
High	29 (16.8%)
Middle	34 (19.7%)
Low	32 (18.5%)
Missing	78 (45.1%)
Subtype	
Adenocarcinoma	155 (89.6%)
Squamous cell carcinoma	10 (5.8%)
Large cell carcinoma	1 (0.6%)
Other	7 (4.0%)

### Mutation profiling

**Figure 1:**
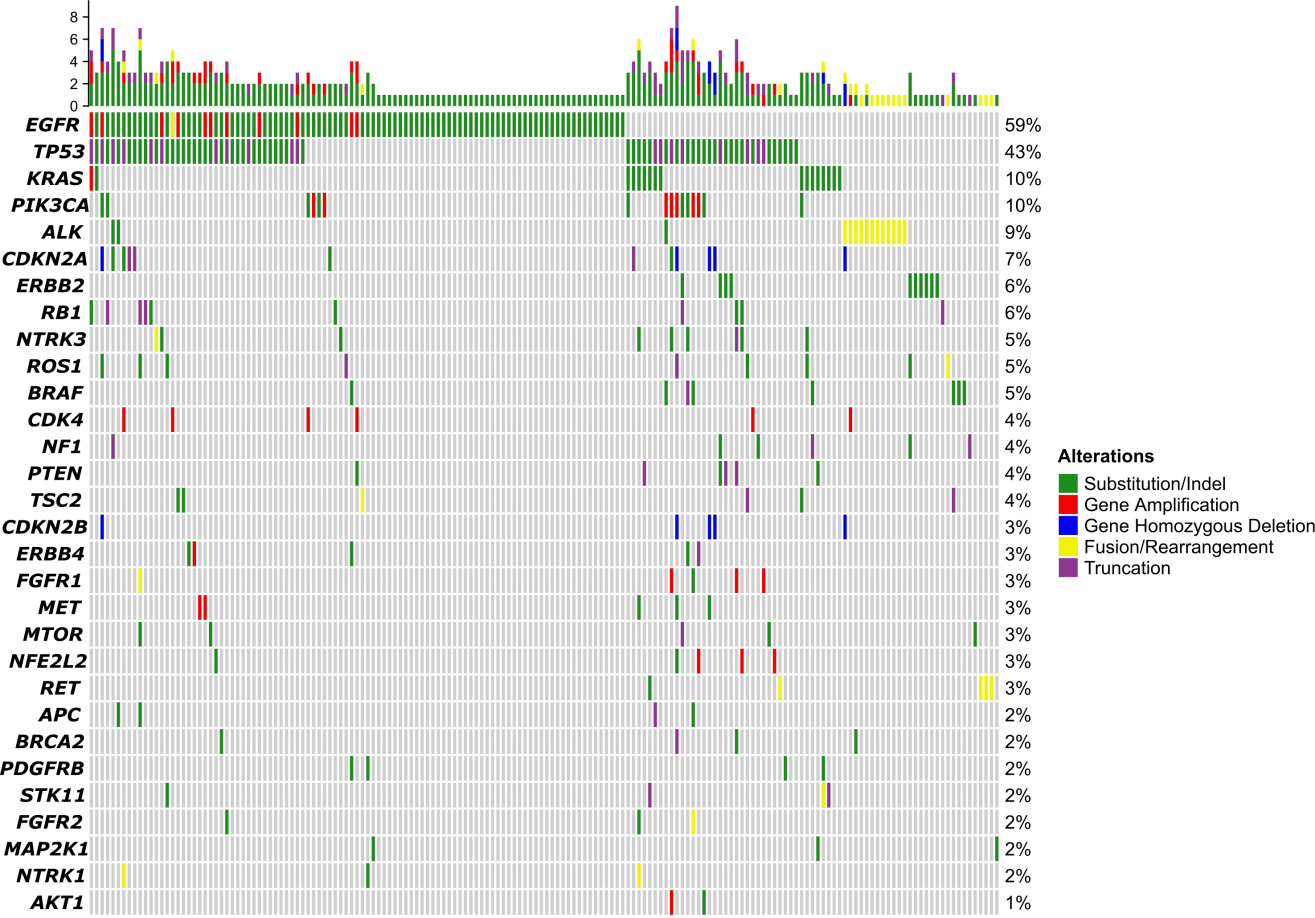
Gene mutation profiling.

Of the 173 patients, 168 had qualified samples for sequencing. As shown in **Figure 1**, the top 5 mutated genes were *EGFR*, *TP53*, *KRAS*, *PIK3CA*, and *ALK*. The mutation frequency of *EGFR* was 59%. Most of them were substitution or short insert/deletion (indel), and only 11 patients (7%) had *EGFR* amplification. Pathway enrichment analysis indicated that genes with mutations in more than 1% of patients were mostly related to the regulation of protein phosphorylation, regulation of kinase activity, and regulation of cell proliferation (**Supplementary Figure 1A**). We also compared the mutation frequency to the MSK cohort. The GMU cohort had a higher percentage of *EGFR* but a lower percentage of *TP53*, *KRAS*, *NF1*, and *STK11* (**Supplementary Figure 1B**).

### Driver genes were associated with tumor grades

**Figure 2:**
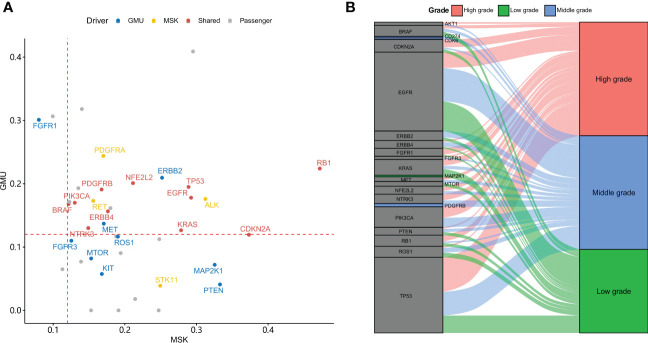
Driver genes in the GMU and MSK cohorts. **(A)** The allele frequency of driver gene mutation in both MSK and GMU cohorts is compared. **(B)** Driver genes preferentially mutate in different tumor grades.

To find out significantly mutated genes, MutSigCV was used. It found 23 and 15 significantly mutated genes in the GMU and MSK cohorts, respectively. The median allele frequency in GMU and MSK cohorts is plotted in **Figure 2A**. Eleven genes were both significantly mutated in the GMU and MSK cohorts. The shared significant genes (*RB1*, *TP53*, *EGFR*, *BRAF*, *PIK3CA*, etc.) had a high allele frequency in both cohorts. *ALK*, *PDGFRA*, *ERBB2*, *RET*, and *MET* had higher alleles in both cohorts but were only significantly mutated in the MSK cohort. *RB1*, *TP53*, and *PIK3CA* were most mutated in high-grade tumors and *EGFR* was most mutated in middle- and low-grade tumors (**Figure 2B**).

### Grade-associated gene mutations and pathway

**Figure 3:**
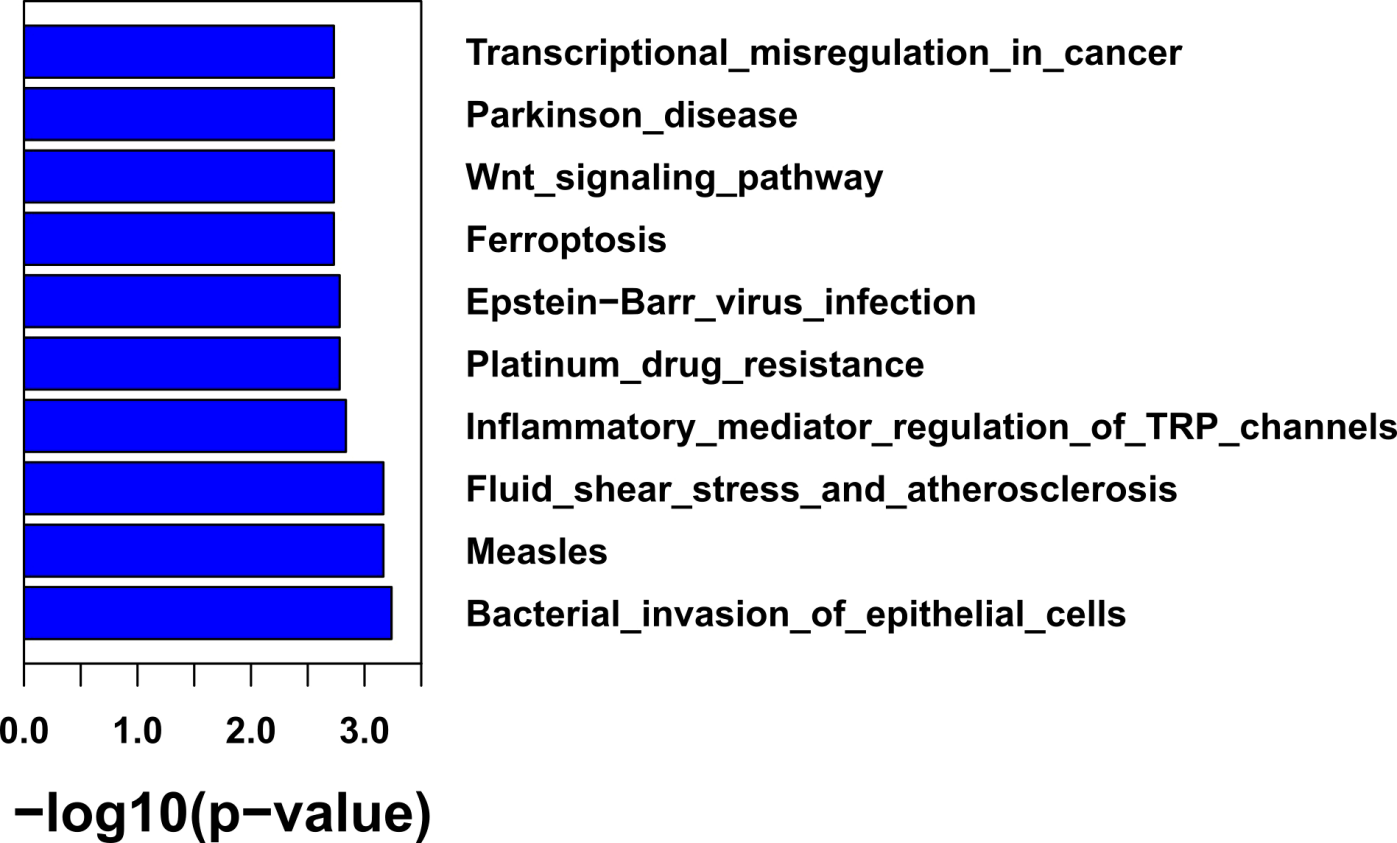
The functional enrichment of genes with mutations in high-grade tumors.

Of the 168 samples sequenced, 95 samples have histological grade records. Gene mutations within low-grade, middle-grade, and high-grade tumors were compared with Fisher’s exact test. The enriched pathways in each grade of tumors were also inferred. When comparing mutation frequency between histological grades (**Supplementary Figures 2A–C**), we found that high-grade tumors had an elevated percentage of mutations in *TP53* (75.9% *vs* 34.4%, *p* = 1.86e-3) and *PIK3CA* (24.1% *vs*. 0%, *p* = 3.58e-3) compared to low-grade tumors (**Supplementary Figure 2C**). Pathway analysis found that high-grade tumors were enriched with mutations in bacterial invasion of epithelial cells (31% *vs*. 0%, *p* = 5.8e-4), Epstein–Barr virus (EBV) infection (79.3% *vs*. 37.5%, *p* = 1.72e-3), and the Wnt signaling pathway (75.9% *vs*. 34.4%, *p* = 1.91e-3) (**Figure 3**). No gene was significantly mutated between low- and middle-grade tumors and between middle- and high-grade tumors (**Supplementary Figures 2A, B**). 

### High-grade tumors were associated with Epstein–Barr virus-related gene mutations

**Figure 4:**
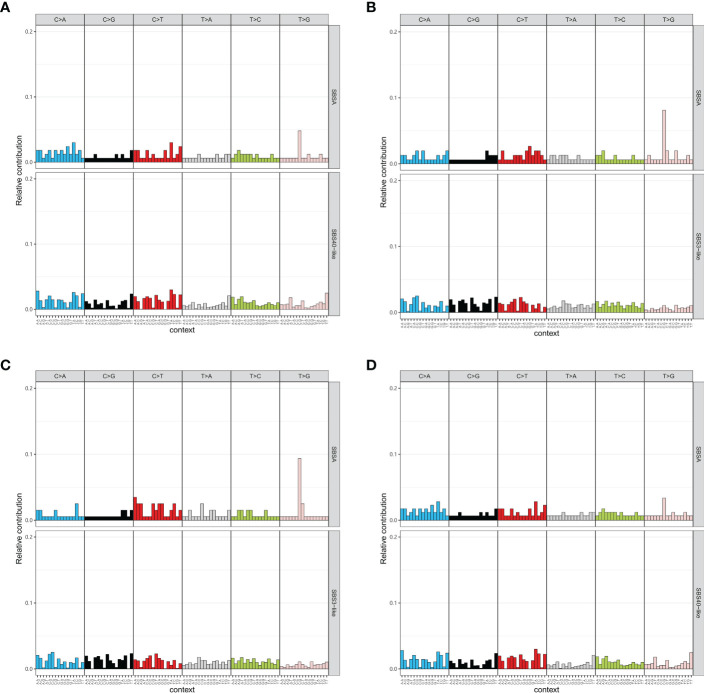
Mutational signature of tumors with different grades. The mutational signatures of high-grade tumors **(A)**, middle-grade tumors **(B)**, and low-grade tumors **(C)** are compared to COSMIC signatures. **(D)** Tumors with mutations in the Epstein–Barr virus-related pathway have a signature similar to COSMIC SBS40.

Mutational signature analysis indicated that high-grade tumors only had one mutational signature, which is like SBS40 (cosine similarity = 0.78, **Figure 4A**). Middle-grade and low-grade tumors each had a mutational signature like SBS33 with cosine similarities of 0.69 and 0.64, respectively (**Figures 4B, C**). Genes with mutations in the EBV infection pathway had a similar mutation profile to COSMIC SBS40 (cosine similarity = 0.81) (**Figure 4D**). We also compared the mutation profile of genes with mutations in the EBV infection pathway for the MSK cohort to the COSMIC signature database. Those tumors also presented mutational signatures like SBS40 (cosine similarity = 0.78) (**Supplementary Figure 3A**). The mutational signature similarity between GMU and MSK cohorts was 0.76. SBS40 was considered related with age in the COSMIC database. We failed to find an association between age and tumor grade (**Supplementary Figure 3B**).

### Actionability and tumor mutational burden

**Figure 5:**
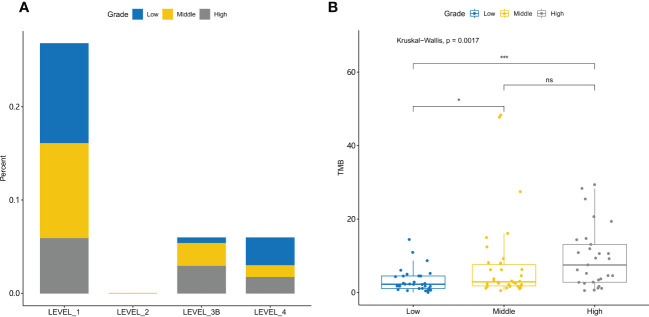
Actionability and tumor mutational burden are different for low-, middle-, and high-grade tumors **(A)** The evidence levels of actionability in lung cancers are shown for different grades of tumors. **(B)** Tumor mutational burden (TMB) is significantly different among different grades of tumors. *, p-value<0.05; ***, p-value<0.001; ns, Non-significant.

Gene mutations are regularly used as biomarkers for targeted therapy and immunotherapy. We further compared the actionability among different grades of tumors (**Figure 5A**). High-grade tumors had a lower percentage of high actionability evidence (Level_1). High-grade tumors had fewer *EGFR* actionable mutations than the other tumors (**Supplementary Figures 4A–C**).

In high-grade tumors, tumors with *FGFR1* or *TP53* mutations had higher TMB than those without, and tumors with *EGFR* mutations had lower TMB (**Supplementary Figure 4D**). In middle-grade tumors, *PDGFRB*- and *PIK3CA*-mutated tumors had a higher TMB, and *EGFR*-mutated tumors had a lower TMB (**Supplementary Figure 4E**). In low-grade tumors, *TP53*-mutated tumors had a higher TMB (**Supplementary Figure 4F**).

TMB is a biomarker for immunotherapy. TMB was significantly different among different grade tumors (Kruskal–Wallis test, *p*-value = 1.7e-3) (**Figure 5B**). Further *post-hoc* comparison confirmed that TMB in high- and middle-grade NSCLC tumors is significantly higher than that in low-grade tumors with *p*-value = 6.6e-4 and 4.2e-2, respectively.

## Discussion

In this study, we investigated gene mutations in NSCLC. Our analysis of the gene mutation profile in the GMU cohort revealed a different pattern compared to the MSK cohort. Specifically, we found that *EGFR* mutations were more frequent in the GMU cohort (59% *vs*. 10%) than in the MSK cohort (10%), whereas *TP53* (43% *vs*. 66%) and *KRAS* (10% *vs*. 41%) mutations were less common. Our results are consistent with a previous study on NSCLC patients in a Chinese population, which reports a higher percentage of *EGFR* mutations (56%) and a lower percentage of *KRAS* mutations (12%) (15).

Histological grade is an independent prognostic factor for resectable NSCLC (16) and high-grade NSCLC is more dangerous than the low-grade one (17). Understanding the mechanism of high-grade NSCLC could help develop novel treatments for patients. To identify candidate driver genes, MutSigCV was used, and 20 genes were found to be significantly mutated in the GMU cohort. Of those, 11 genes were also significantly mutated in the MSK cohort, including *RB1*, *EGFR* (15, 18), *TP53*, and *PIK3CA* (19). All the sharing candidate driver genes had a higher variant allele frequency in both cohorts, and *RB1*, *TP53*, and *PIK3CA* mutations were more commonly detected in high-grade NSCLC. *TP53* mutations were found to occur more frequently in high-grade ovarian (20) and endometrial cancers (21), but not in breast cancer (22). *RB1* mutations were found to be prone to high-grade bladder cancer (23), astrocytic gliomas (24), and Merkel cell carcinoma (25), and they could function by activating the *NF-κB* pathway and exerting anti-inflammatory effects (26). Furthermore, *EGFR*-resistant lung adenocarcinoma with *RB1* mutations could transform into a more aggressive small-cell lung cancer (27). Similarly, loss of *RB1* and *TP53* in NSCLC could also lead to the transformation of NSCLC into small-cell lung cancer (28). The association of *RB1*, *TP53*, and *PIK3CA* mutations with histological grade in NSCLC could be meaningful for predicting histological grade in certain scenarios, such as using ctDNA for gene mutation detection and histological grade prediction before surgery.

Further pathway enrichment analysis of differentially mutated genes revealed that the bacterial invasion of epithelial cells pathway and EBV infection pathway were significantly mutated in high-grade NSCLC. EBV has been shown to promote tumor development in many cancers, including lung cancer (29, 30), breast cancer (31), gastric cancer (32), and nasopharyngeal carcinoma (33). In NSCLC, EBV infection has been closely linked to pulmonary lymphoepithelioma-like carcinomas (a subtype of large cell carcinoma) (29, 30), lung squamous-cell carcinomas (34), and lung adenocarcinomas (35). EBV has also been associated with a higher mutation rate in the NF-κB pathway, including *TRAF3* and *NFKBIA* (30, 36). Notably, gene mutations in the EBV infection response pathway, such as *TRAF3* (37), *NFKBIA* (30), *RB1* (38), and *PIK3CA* (39), have been reported to play an important role during cancer development. Mutations in the EBV infection pathway could potentially be a mechanism by which EBV evades the immune response in hosts.

Previous studies have reported an association between EBV infection and histological grade in lymphoma (40) and breast cancer (41), but this association has not been explored in lung cancer. To further investigate, mutational signature analysis was conducted, revealing that high-grade NSCLC exhibited a mutational signature similar to COSMIC SBS40, which is potentially related with age. To assess the influence of stages, the samples were divided into early stage (Stages 0, I, and III) and advanced stage (Stages III and IV) groups, and the mutational signatures of high-grade tumors were evaluated. Interestingly, both groups exhibited only one mutational signature, which was similar to COSMIC SBS40, with corresponding cosine similarities of 0.77 and 0.81 (**Supplementary Figures 5A, B**). The majority of the samples in this study (89%) were adenocarcinoma; thus, further analysis focused on adenocarcinoma samples. The only mutational signature identified in high-grade adenocarcinoma samples was also similar to COSMIC SBS40 (cosine similarity = 0.77) (**Supplementary Figure 5C**). Further analysis of the genes with mutations in the EBV infection pathway revealed that their mutational signature was also similar to COSMIC SBS40, with a cosine similarity of 0.69. To validate these findings, an independent cohort (MSK) was used, and the mutational signature of the genes in the EBV pathway was found to be similar to COSMIC SBS40, with a cosine similarity of 0.78. Between the GMU and MSK cohorts, the mutational signatures had a cosine similarity of 0.76, indicating mostly similar signatures to COSMIC SBS40. The etiology of COSMIC signature SBS40 is unknown, but it could potentially be related with age, as suggested by the COSMIC database. Our results suggest that SBS40 may also be related to EBV infection, possibly due to the continuous damage caused by the virus to lung tissue, leading to the formation of high-grade lung tumors with aging.

Targeted therapy and immunotherapy are commonly used in the treatment of lung cancer (42). Understanding the treatment efficacy of high-grade cancer could aid in the development of novel therapies. Our analysis of targeted genes revealed that high-grade NSCLC has a low percentage of actionable genes with LEVEL_1 evidence, and *EGFR* mutations are the most actionable with LEVEL_1 evidence. However, the lower percentage of *EGFR* mutations in high-grade NSCLC results in its low actionability. Despite the lower percentage of targeted therapy treatment, high-grade NSCLC may benefit from immunotherapy. TMB is a biomarker of immunotherapy, and high TMB is associated with a better prognosis (43, 44). High-grade NSCLC has a higher TMB value than low- and middle-grade NSCLC, which suggests that it may respond better to immunotherapy. Furthermore, in high-grade NSCLC, TMB is positively correlated with *TP53* and *FGFR1* mutations and negatively correlated with *EGFR* mutations, indicating that these genes could be used to predict the TMB value in high-grade NSCLC.

One limitation of this study is that it only analyzed 450 cancer-related genes, which may have precluded the identification of other related genes or functions. Additionally, the association between EBV infection status and high-grade NSCLC requires further investigation to validate the finding that the mutational signature of EBV infection is associated with high-grade NSCLC in this cohort and the MSK cohort. Future studies should analyze the association between EBV infection status and high-grade NSCLC.

In summary, our analysis revealed the defected pathway bias in different grades of NSCLC and demonstrated a possible association between EBV infection and high-grade NSCLC. We also found that the current targeted therapy needs more attention in treating high-grade NSCLC, while immunotherapy may be a promising approach for its treatment.

## Data availability statement

The original contributions presented in the study are included in the article/**Supplementary Material**. Further inquiries can be directed to the corresponding authors.

## Ethics statement

The studies involving human participants were reviewed and approved by the Ethics Committee of the Affiliated Hospital of Guilin Medical University. The patients/participants provided their written informed consent to participate in this study. 

## Author contributions

MZ, YZ, and LL designed the research. YW, SQ, YL, LY, MZ, YZ, and LL performed data collection and analysis. YW, SQ, and YL wrote the manuscript draft. YW, SQ, YL, MZ, YZ, and LL prepared the manuscript. All authors contributed to the article and approved the submitted version.

## Supplementary material

Supplementary Figure 1 Mutation profile of MSK cohort **(A)** Log-transformed P-value is shown for the functional enrichment of the top 30 mutated genes. **(B)** The gene mutation frequency is compared between the MSK and GMU cohorts.

Supplementary Figure 2 Mutational frequency is compared among different grades of tumors **(A–C)** The frequency of mutated genes is compared within low-, middle-, and high-grade tumors.

Supplementary Figure 3 The mutational signature of Epstein-Barr virus-related genes in the GMU cohort is compared to that in the MSK cohort. **(A)** The mutational signatures of Epstein-Barr virus-related genes in the GMU and MSK cohorts are compared to COMIC SBS40. **(B)** The age distribution shows no difference within low-, middle-, and high-grade lung tumors.

Supplementary Figure 4 Actionability of mutations from tumors of different grades **(A–C)** The distribution of evidence levels in high-, middle-, and low-grade tumors is shown. **(D–F)** Tumor mutational burden (TMB) is compared between tumors with and without mutations in high-, middle-, and low-grade tumors.

Supplementary Figure 5 Influence of histological subtypes and stages on high-grade tumors Mutational signatures are shown for lung adenocarcinoma **(A)**, and early b and advanced-stage **(C)** tumors.
