# Supplementary figures and images for: Tumor grade-associated genomic mutations in Chinese patients with non-small cell lung cancer
Source: Front Oncol. 2023 Mar 20;13:1119575. doi: 10.3389/fonc.2023.1119575 (PMC10067928; doi:10.3389/fonc.2023.1119575)

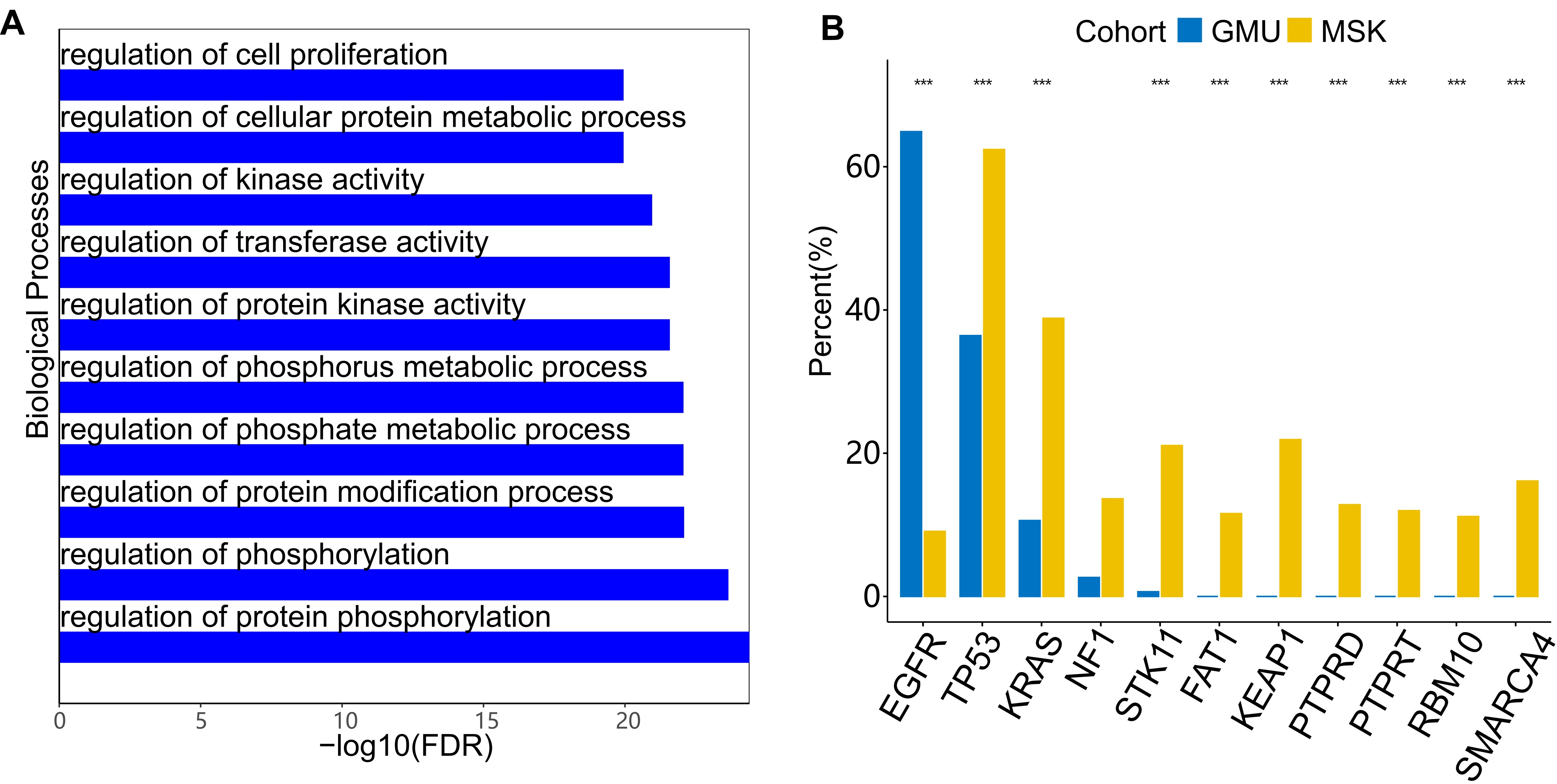

Supplement: Supplementary Figure 1 — Mutation profile of MSK cohort (A) Log-transformed P-value is shown for the functional enrichment of the top 30 mutated genes. (B) The gene mutation frequency is compared between the MSK and GMU cohorts. [file Image_1.jpeg]

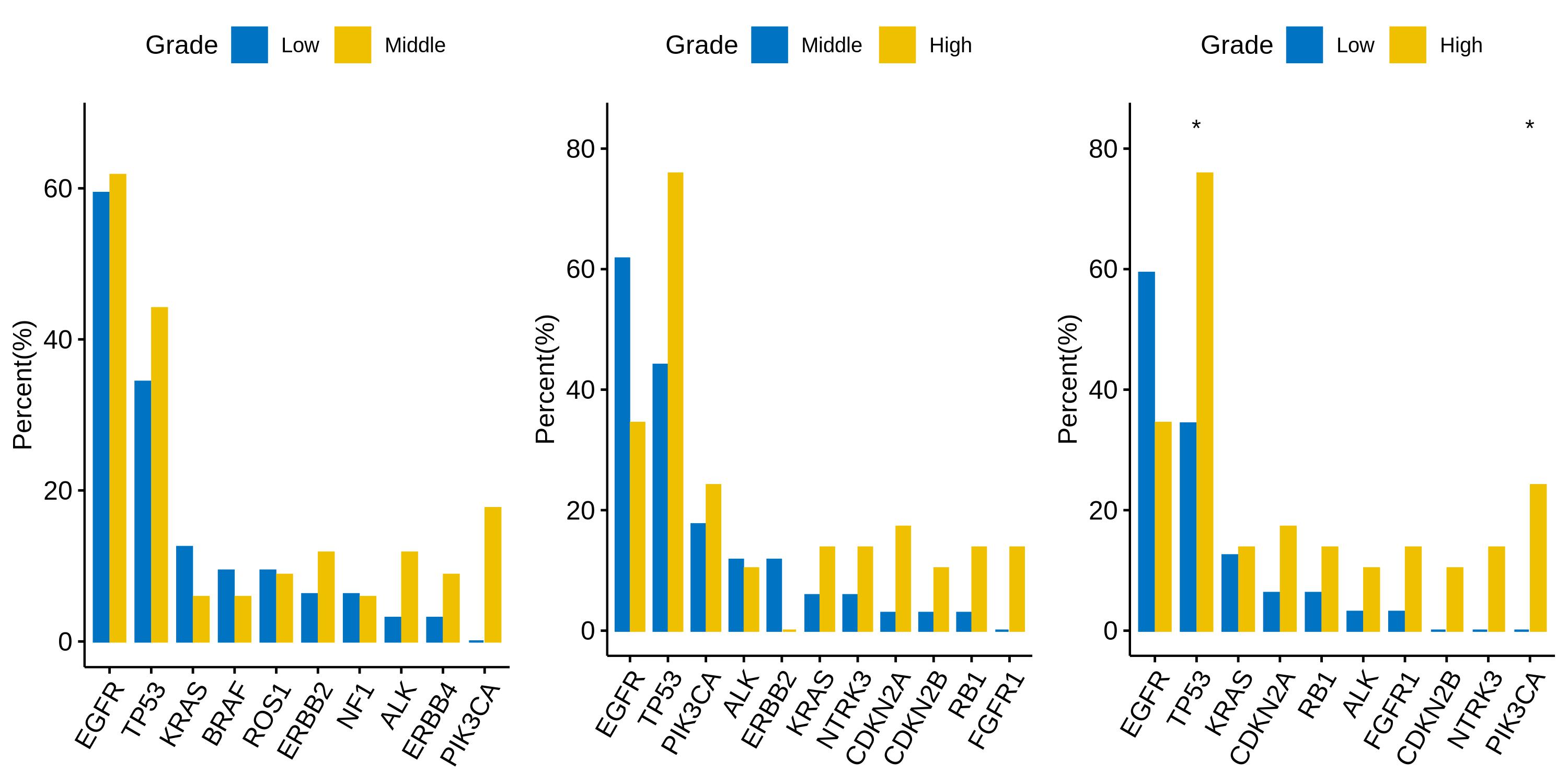

Supplement: Supplementary Figure 2 — Mutational frequency is compared among different grades of tumors (A–C) The frequency of mutated genes is compared within low-, middle-, and high-grade tumors. [file Image_2.jpeg]

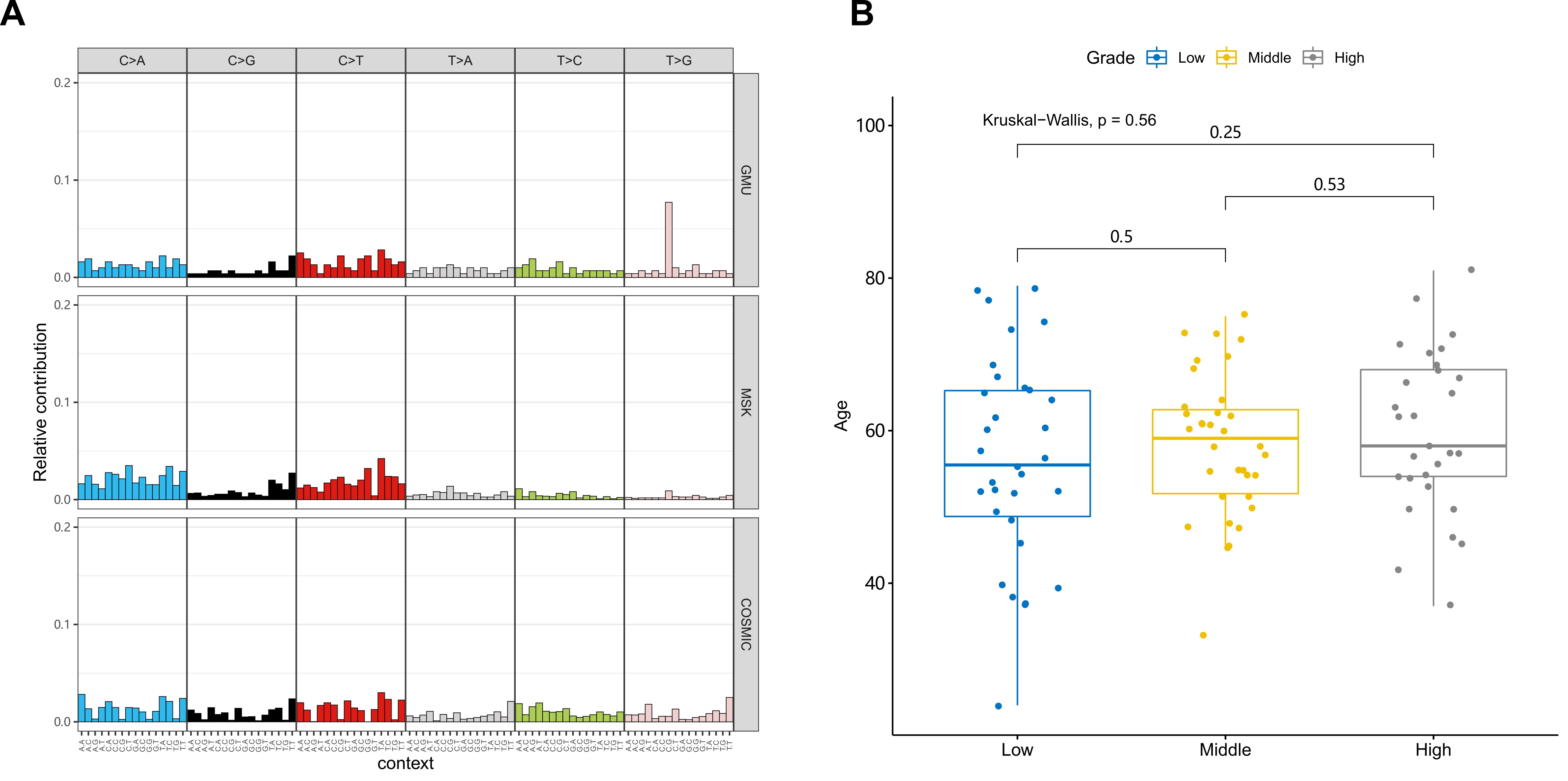

Supplement: Supplementary Figure 3 — The mutational signature of Epstein-Barr virus-related genes in the GMU cohort is compared to that in the MSK cohort. (A) The mutational signatures of Epstein-Barr virus-related genes in the GMU and MSK cohorts are compared to COMIC SBS40. (B) The age distribution shows no difference within low-, middle-, and high-grade lung tumors. [file Image_3.jpeg]

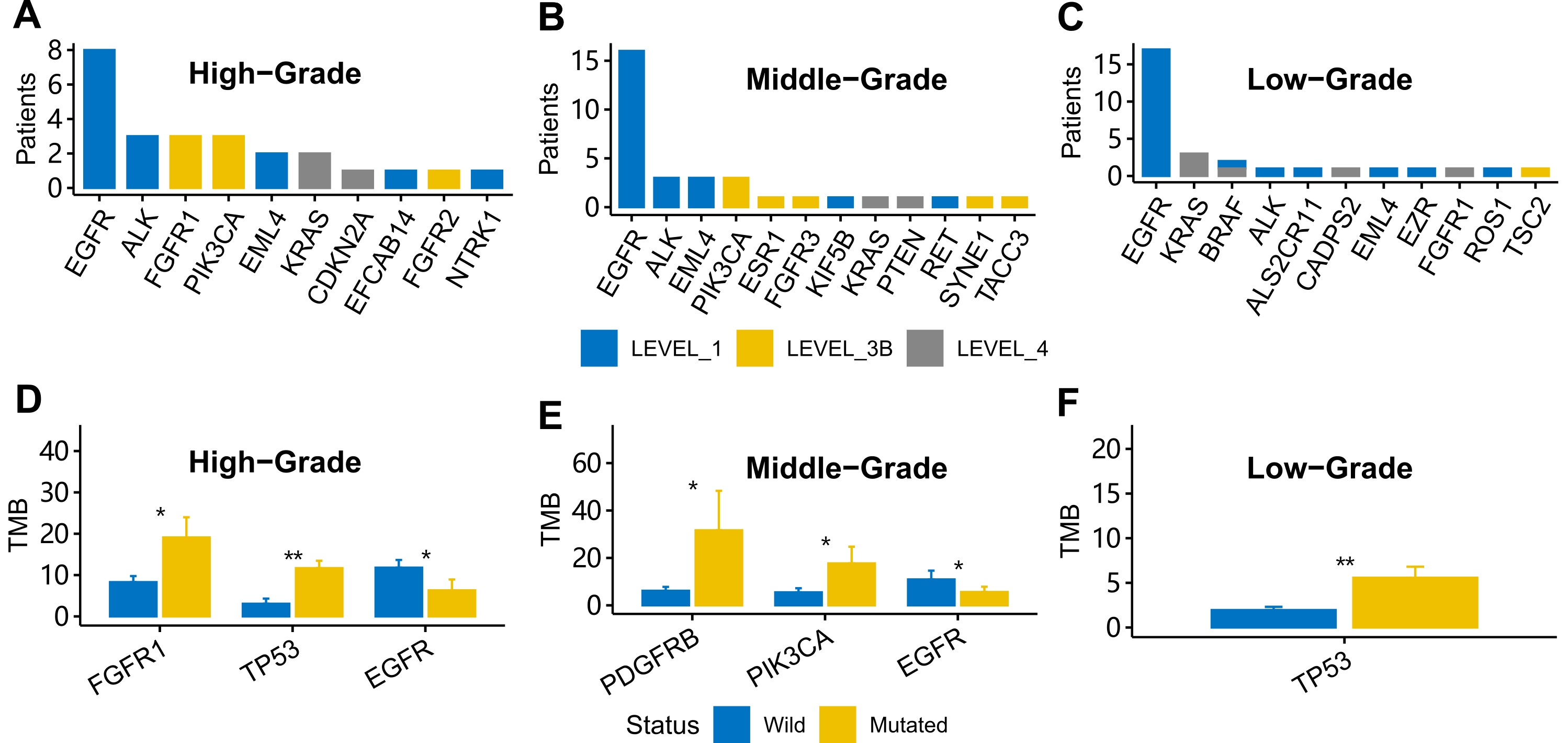

Supplement: Supplementary Figure 4 — Actionability of mutations from tumors of different grades (A–C) The distribution of evidence levels in high-, middle-, and low-grade tumors is shown. (D–F) Tumor mutational burden (TMB) is compared between tumors with and without mutations in high-, middle-, and low-grade tumors. [file Image_4.jpeg]

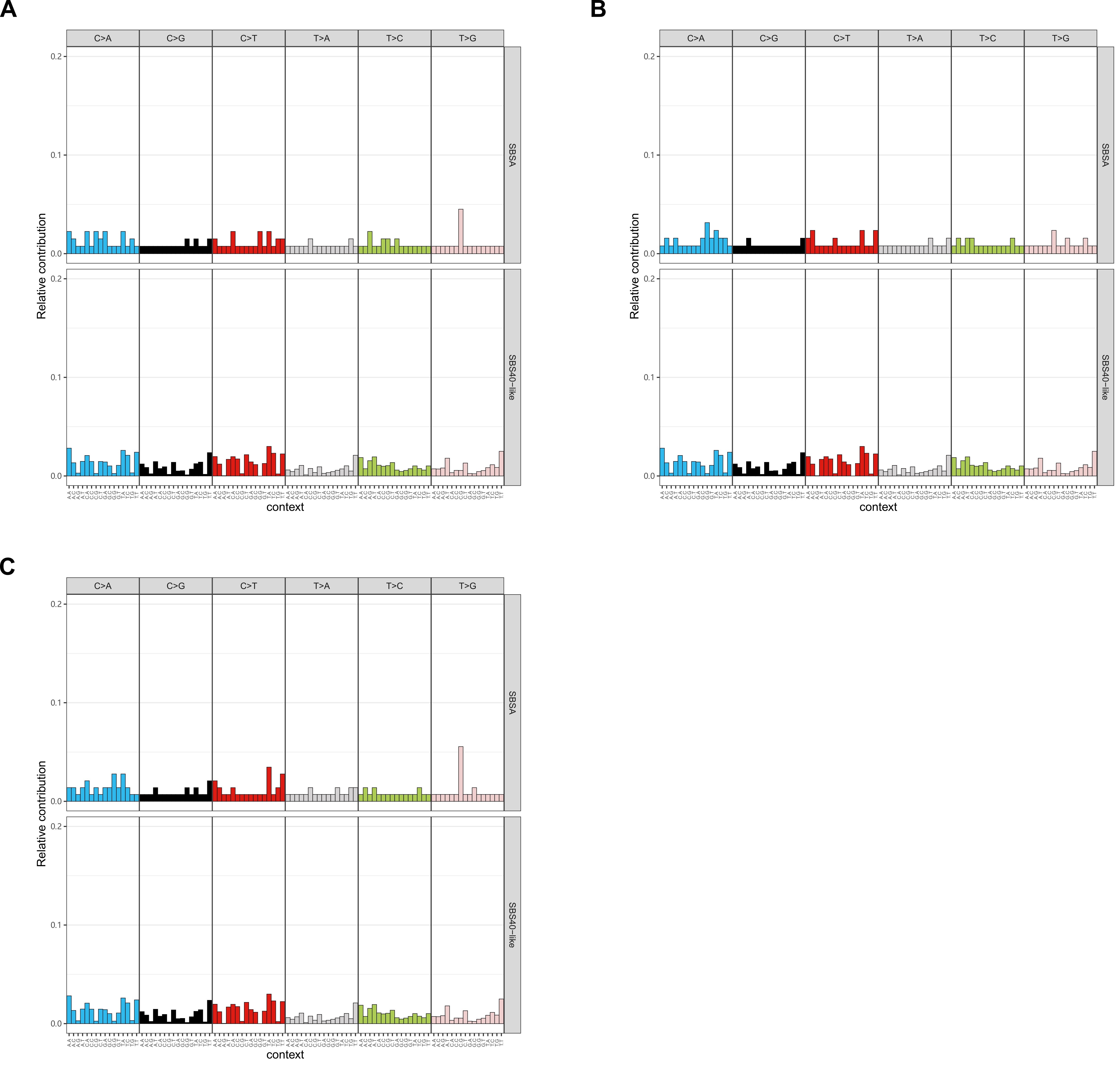

Supplement: Supplementary Figure 5 — Influence of histological subtypes and stages on high-grade tumors Mutational signatures are shown for lung adenocarcinoma (A), and early b and advanced-stage (C) tumors. [file Image_5.jpeg]
